# Supplementary material for: Enhancing the Relative Sensitivity of V5+, V4+ and V3+ Based Luminescent Thermometer by the Optimization of the Stoichiometry of Y3Al5−xGaxO12 Nanocrystals
Source: Nanomaterials (Basel). 2019 Sep 25;9(10):1375. doi: 10.3390/nano9101375 (PMC6836024; doi:10.3390/nano9101375)
Supplement: Supplementary file 1 [file nanomaterials-09-01375-s001.pdf]

nanomaterials

Article

Enhancing the Relative Sensitivity of V^{5+}, V^{4+} and V^{3+} Based Luminescent Thermometer by the Optimization of the Stoichiometry of $Y_3Al_{5-x}Ga_xO_{12}$ Nanocrystals

Karolina Kniec *, Karolina Ledwa and Lukasz Marciniak *

Institute of Low Temperature and Structure Research, Polish Academy of Sciences, Okólna 2, 50-422 Wroclaw, Poland; k.ledwa@intibs.pl

* Correspondence: k.kniec@intibs.pl (K.K.); l.marciniak@intibs.pl (L.M.)

Received: 17 August 2019; Accepted: 24 September 2019; Published: date

Appendix A

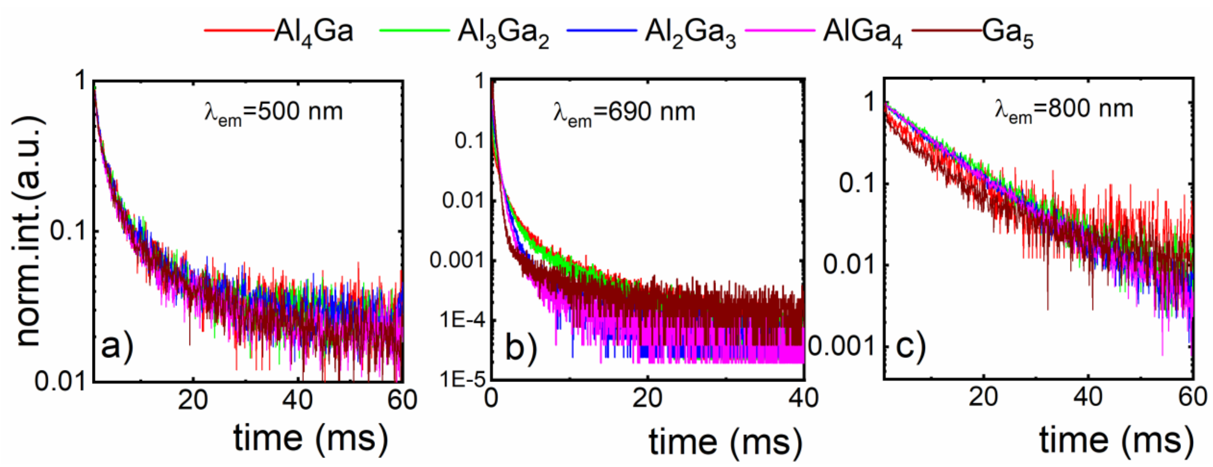

Figure S1. (**a**), (**b**), (**c**) The luminescence decay profile of V^{5+}, V^{4+} and V^{3+} ions for different Ga^{3+}, respectively.

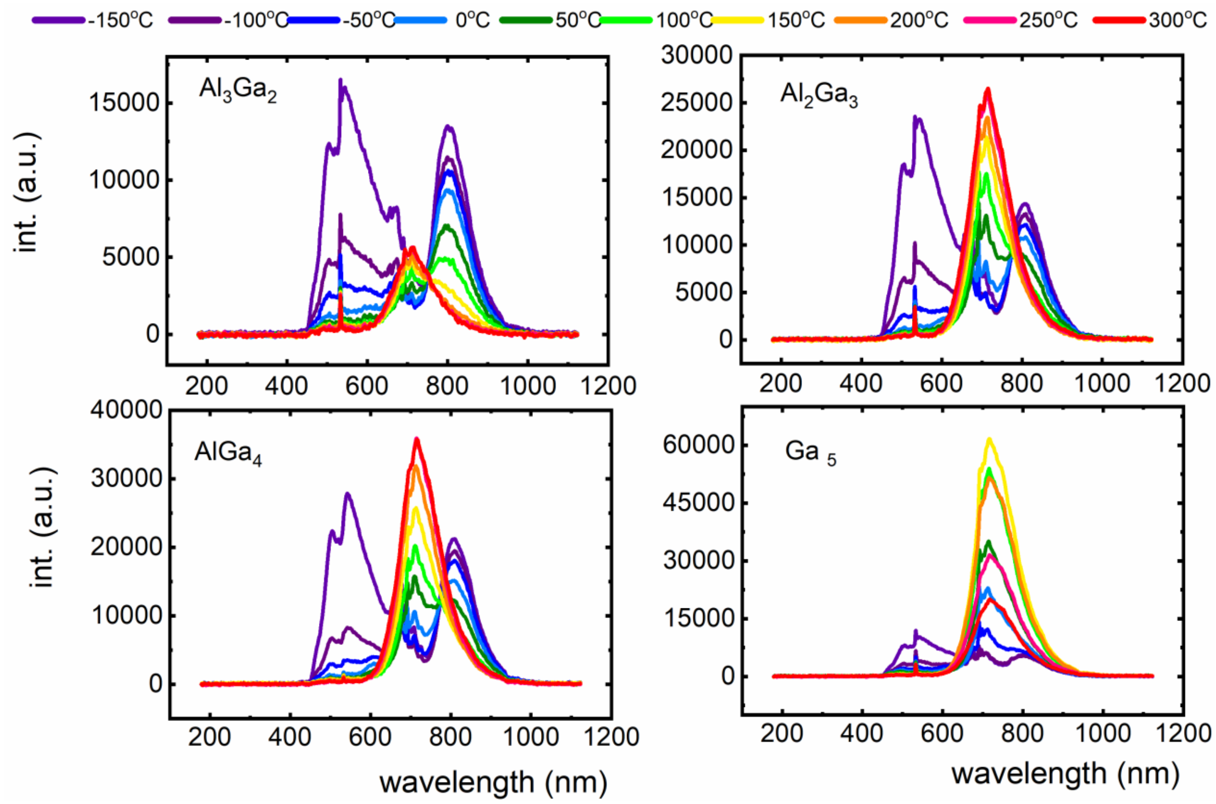

Figure S2. Emission spectra of V-doped nanocrystals recorded in the range of −150°C–300°C.
